# Development and validation of a duplex real-time PCR method for the simultaneous detection of celery and white mustard in food

**DOI:** 10.1186/2045-7022-1-S1-O24

**Published:** 2011-08-12

**Authors:** Margit Cichna-Markl, Magdalena Fuchs, Rupert Hochegger

**Affiliations:** 1 University of Vienna, Department of Analytical Chemistry, Vienna, Austria; 2 Austrian Agency for Health and Food Safety, CC Biochemistry, Vienna, Austria

## 

Celery (celery root: Apium graveolens var. Rapaceum; leaf celery: A. g. var. Secalinum; celery stalks: A. g. var. Dulce) and mustard (white or yellow mustard: Sinapis alba; black mustard: Brassica nigra; brown or oriental mustard: Brassica juncea) are frequently used as ingredients in sauces, spices, sausages and other meat-products as well as in convenience products. Within the European Union, the presence of potentially allergenic celery and mustard in foodstuffs has to be declared according to the EU legislative 2007/68/EC. The aim of the present study was to develop and validate a duplex real-time PCR method allowing the simultaneous detection of traces of celery and white mustard in food. Primers and TaqMan probes were designed for the Apium graveolens NADPH-dependent mannose-6-phosphate reductase mRNA as well as the Sinapis alba mRNA for MADS D protein. PCR was performed on the RotorGene RG-3000 from Corbett Life Sciences. With the optimized duplex assay DNA extracted from celery root, leaf celery and celery stalks as well as DNA from white mustard was amplified. The assay did not show any cross-reactivity with more than 60 food matrices, among them important members of the plant families Apiaceae and Brassicaceae, spices and different meat species.

The LOD in serially diluted DNA extracts from celery root, leaf celery, celery stalks and white mustard was found to be 2 pg/μL (10 pg absolute). The PCR efficiency was 99.4% for celery root, 108.3% for celery stalks, 96.5% for leaf celery and 99.0% for white mustard. The LOD in DNA extracts obtained by extraction of model sausages was 0.005% (50 mg/kg) for celery and 0.001% (10 mg/kg) for white mustard, in both raw and brewed model sausages. The PCR efficiency was 90.0% (celery) and 101.1% (white mustard) in raw model sausages and 85.8% (celery) and 91.2% (white mustard) in brewed model sausages.

